# Effects of a polyherbal formulation on inflammation and histopathological alterations in mice with ovalbumin-induced allergic asthma 

**DOI:** 10.22038/AJP.2022.20050

**Published:** 2022

**Authors:** Maryam Hamzeloo-Moghadam, Seyyed Shamsadin Athari, Hanieh Kashafroodi, Tahereh Dargahi, Leila Ara, Rasool Choopani

**Affiliations:** 1 *Traditional Medicine and Materia Medica Research Center and Department of Traditional Pharmacy, School of Traditional Medicine, Shahid Beheshti University of Medical Sciences, Tehran, Iran *; 2 *Department of Immunology, Faculty of Medicine, Zanjan University of Medical Sciences, Zanjan, Iran*; 3 *Traditional Medicine and Materia Medica Research Center, Shahid Beheshti University of Medical Sciences, Tehran, Iran*; 4 *Department of Traditional Medicine, School of Traditional Medicine, Shahid Beheshti University of Medical Sciences, Tehran, Iran*

**Keywords:** Allergic asthma, Balb/C mice, Monzej-e-balgham, Iranian traditional medicine

## Abstract

**Objective:**

Allergic asthma is a complex inflammatory disorder that affects the airways. As an ancient medical system, Iranian Traditional Medicine (ITM) recommends a polyherbal formula called “*Monzej-e-balgham*” for the treatment of asthma. In the present investigation, the antiasthmatic effects of “*Monzej-e-balgham*” were examined in a murine model of allergic asthma.

**Materials and Methods:**

Twenty-eight Balb/c mice weighing 15-20 g were allocated into 4 groups. As negative and positive controls, groups I and II received phosphate-buffered saline (PBS) and ovalbumin (OVA) solutions, respectively. Groups III and IV were first sensitized with OVA and then respectively treated with “*Monzej-e-balgham*” (63 mg/kg) and budesonide. Finally, bronchoalveolar lavage fluid (BALF) and lung tissues of the animals were collected and used for eosinophil counting, Th2 type interleukins (IL-5, IL-13, and IL-33) measurement, and histological examinations.

**Results:**

“*Monzej-e-balgham*” significantly reduced the number of eosinophils and the levels of IL-5, IL-13, and IL-33 in BALF specimens compared to OVA-sensitized group (p<0.05). It also ameliorated histopathological changes of the lung tissues such as goblet cells hyperplasia and mucus overproduction in comparison to group II. Interestingly, the results of the “*Monzej-e-balgham*”-treated group were comparable with those obtained for budesonide-inhaled mice.

**Conclusion:**

The present data indicated a mechanism that involves Th2 inflammatory responses in allergic asthma and suggested a polyherbal mixture for the treatment of this disease.

## Introduction

Allergic asthma is a complicated disease that triggers an inflammatory process in the respiratory tract by increasing migration of leucocytes, especially eosinophils, into the lung tissue, resulting in an abnormal function of the respiratory system. This condition causes inflammation-induced obstruction of the airways and changes in lung tissues (Bochner et al., 2013 ▶). Asthma-related symptoms include shortness of breath, coughs, and wheezing during exhale (Kave et al., 2013 ▶). The number of patients who suffer from allergic asthma is increasing in the world and this causes a critical challenge for human health (Braman, 2006 ▶). Asthma affects more than 300 million people around the world and estimates show that this will increase to 400 million patients by 2025. This disease can occur in any age. The incidence rate of asthma among boys, before the age of 12, is double that among girls but this rate is equal in adults (Fuseini and Newcomb, 2017 ▶). The common cure for asthma includes inhalation of β2-agonists and glucocorticoids (Choi et al., 2013 ▶). These medications are associated with various side effects such as restlessness, drowsiness, tremor, dizziness, dry mouth, anorexia, and nausea and they are short-lasting and expensive (Stoloff and Kelly, 2011 ▶). Oxidative stress may worsen airway inflammation in bronchial asthma via inducing various inflammatory cytokines, increasing bronchial hyperresponsiveness, causing bronchospasm, and enhancing mucin secretion (Cho and Moon, 2010 ▶). Animal models of allergic asthma also indicated that the airways and vascular system face elevated reactive oxygen species and lipid peroxides levels during the disease onset, leading to inflammation in these tissues (Al-Harbi et al., 2015 ▶). However, direct contribution of oxidative stress to asthma pathology remains at least somewhat controversial, because antioxidant effects on alleviation of asthma complications have proven rather unsuccessful (Jesenak et al., 2017 ▶).

Nowadays, many people are showing great attention to complementary and alternative medicine for the treatment of a variety of diseases. Due to the chronic nature of respiratory disorders and the lack of definitive treatments, people are looking for alternative therapies with fewer side effects and longer-lasting benefits (Ko et al., 2006 ▶; Nakano et al., 2012 ▶; Slader et al., 2006 ▶).

In Iranian traditional medicine (ITM), the term “*Rabv*” is closely associated with asthma and its symptoms are very similar to this respiratory disease. The phlegmatic type of this disease is termed “*Rabve-e-balghami*”, which comprises the most commonly occurring type of asthma and is accompanied by thick phlegm accumulating in the respiratory tract (Amini et al., 2019 ▶). The medications that can overcome this condition by dissolving the thickly formed phlegm, can be of great importance in asthma therapy. 

“*Monzej-e-balgham*” is a polyherbal mixture that is traditionally used to dissolve phlegm and consists of *Glycyrrhiza glabra* L., *Rosa *x* damascena *Herrm., *Adiantum capillus-veneris* L., *Onopordum acanthium* L., *Vitis vinifera* L., *Ficus carica* L. and *Foeniculum vulgare *Mill (Arzani, 2002 ▶). In the present study, a polyherbal formula that has been recommended by Arzani (consisted of large raisin, anise, liquorice, cotton thistle, maiden hair, fig and damask rose), (Arzani, 2002 ▶) was evaluated in a mouse model of asthma. 

## Materials and Methods

**Chemicals**

Ovalbumin (OVA) and aluminum hydroxide (Sigma-Aldrich, USA), urethane (Sigma-Aldrich, USA), Mouse IL-5 and IL-13 ELISA kits (R&D Systems, Minneapolis, MN, USA), TRIzol (Invitrogen, USA), cDNA synthesis kit (Thermo Scientific, USA), Rotor-Gene Q thermal cycler (Qiagen, Germany), budesonide (Cipla, India), methanol, Folin-Ciocalteu reagent and pyrogallol (Merck, Germany) were used in the present study.

**Plant material**

All plant materials of the traditional formulation were provided from Tehran local market (2018) and their identity was confirmed by botanists at the Traditional Medicine and Materia Medica Research Center (TMRC), Shahid Beheshti University of Medical Sciences, Tehran, Iran. The voucher specimens were deposited at TMRC Herbarium for future reference. As has been recommended in the traditional references (Arzani, 2002 ▶; Rezghi et al., 2021 ▶), a mixture of seven plants including *Vitis venifera *L. (Large raisin, 5 g), *Pimpinella anisum* L. (Anise, 5 g), *Glycyrrhiza glabra* L. (Liquorice, 9.5 g), *Onopordum acanthium* L. (Cotton thistle, 6.3 g), *Adiantum capillus veneris* L. (Maiden hair, 15.9 g), *Ficus carica* L. (Fig, 32 g) and *Rosa damascena *L. (Damask rose, 9.5 g) (voucher numbers HMS-522-528, respectively), which is called “*Monzej-e-balgham*”, was provided. The mixture was first boiled in water (1:10 w/v) for 1 hr and cooled at room temperature and then the extract was filtered and freeze-dried. The extraction yield was 19%.

**Total phenolics content**

The British Pharmacopeia method was followed to determine the total phenolic content considering the Folin-Ciocalteu method (Hasanloo et al., 2011 ▶; Soodi et al., 2016 ▶) with pyrogallol (concentrations, 0.25-0.0312 mg/ml in two fold dilution) as a standard solution. The reaction mixture was provided by combining 2 ml of the methanolic solution of the extract (8 mg/ml), distilled water (10 ml), and Folin-Ciocalteu reagent (1 ml). The mixture volume was adjusted to 25 ml with sodium carbonate solution (29%). After 30 min incubation at room temperature, the absorbance of the solution was read at 760 nm. All experiments were performed in triplicate. The total phenolics content was expressed as pyrogallol equivalent in g per 100 g of dried extract.

**Total tannins content**

Hide powder was used for determining tannin content of the extract (Nikmanesh and Mohammadi-Motamed, 2019). The powder was added to 10 ml of the extract solution. After an hour of shaking, the mixture was filtered and the Folin-Ciocalteu method was performed on 2 ml of the filtrate. The absorbance was measured at 760 nm and the content of total phenols, which was not absorbed by hide powder, was measured using the pyrogallol calibration curve. Total tannin content was calculated by subtracting the non-tannin content from the total phenolic content. The experiments were carried out in triplicate.

**Thin layer chromatography (TLC) fingerprinting of the polyherbal mixture**

Thin Layer Chromatography of the mixture was performed on silicagel plates using ethyl acetate: formic acid: glacial acetic acid: water (100:11:11:26) as the mobile phase and natural product as the reagent. The spots were then evaluated under UV at 366 nm. Each plant and the polyherbal mixture extract powder were dissolved in methanol and the corresponding fingerprint was evaluated.

**Animals**

The present study was approved by the Ethical Committee of Shahid Beheshti University of Medical Sciences (Approval code: IR.SBMU.RETECH.REC.1397.500); the whole work was done in accordance with the NIH Guide for the care and use of laboratory animals. Twenty-eight male Balb/c mice, 6-8 weeks of age weighing 20-15 g, were provided from the Pasteur Institute of Iran. Animals were kept in the animal lab of the School of Traditional Medicine, Shahid Beheshti University of Medical Sciences, for one week and then, used in the study. The animals were kept at 22-24°C, 45-65% humidity, and 12 hr/12 hr light-dark cycle in a pathogen-allergen-free environment. Standard diet and water were accessible for the mice *ad libitum*. 

The mice were randomly allocated to four groups of seven mice: group I (the negative control group) received phosphate-buffered saline (PBS); group II (the positive control group) was sensitized with ovalbumin (OVA); and groups III and IV were first sensitized with OVA and then, treated with oral gavage of “*Monzej-e-balgham*” (63 mg/kg) and inhalation of budesonide, respectively.

**Induction of asthma**

**Figure 1:**
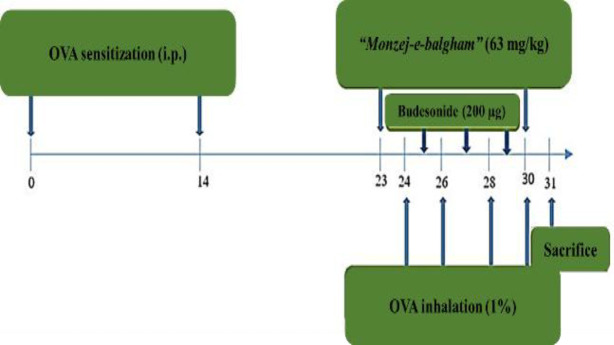
Schematic representation of the experimental design of the present study

Sensitization of the animals was carried out in two distinct steps during one month: First, using intraperitoneal (i.p.) injection of OVA (20 μg/100 μl of physiologic serum and adjuvant, alum) on days zero and 14 and in the second, by nebulization of OVA solution on days 24, 26, 28 and 30. The OVA and PBS solutions were given to the animals by spraying the solution in a way that each day, each animal received 8 mL of OVA 1% in sterile saline solution with the rate of 0.3 ml/min under 0.6 Pas pressure during 20 min. To obtain an optimal dose to be used as a single efficient dose of “*Monzej-e-balgham*”, different concentrations of this medication were evaluated in a pilot study. Consequently, we found that two concentrations of the medication (31.5 and 63 mg/kg) led to more significant results with 63 mg/kg being the most effective dose. Therefore, all mice were treated with 63 mg/kg of the “*Monzej-e-balgham*” on days 24, 26, 28, and 30, 15 min after OVA inhalation and on days 25, 27, and 29 as a single treatment. The mice in group IV received budesonide 5 puffs (200 µg/dose) every 5 min for 20 min on days 25, 27, and 29 (Volovitz et al., 2008). On the 31^st^ day of the experiment, three mice in each group were used for bronchoalveolar lavage fluid (BALF) collection and the remaining four were used for histopathological analysis (Figure 1).

**Table 1:** Primer sequences used in this study

Gene name	Forward primer	Reverse primer
*IL−5*	5'- ATCCAGGAACTGCCTCGTC -3′	5'- ACATTGACCGCCAAAAAGAG -3′
*IL−13*	5'- AATAAGATCAAGAAGAAATGTGCTCAA -3′	5'- GGTCCACACAGGGCAACT -3′
*GAPDH*	5'-GGTCCTCAGTGTAGCCCAAG-3	5'-TGTTCCTACCCCCAATGTGT-3

**Collecting bronchoalveolar lavage fluid** **(BALF) and counting eosinophils**

BALF was obtained through tracheal cannulation after anesthetizing the mice using urethane (1 ml). The resulting samples were kept at -70°C for further measurements (Athari et al., 2016). The number of eosinophils has been determined in BALF specimens of different groups.

**Analysis of Th2 type cytokines expressions and levels**

ELISA method was performed in triplicate to indicate the levels of IL-5, IL-13, and IL-33 in the BALF according to the protocols of an ELISA kit (R&D Systems, Minneapolis, MN, USA) provided by the manufacturer. BALF samples were then used to measure the mRNA expressions of IL-5 and IL-13 using the real time-PCR method. Using TRIzol solution, the total RNA of the BALF samples was extracted and subjected to the cDNA synthesis kit to synthesize the cDNA molecules. Glyceraldehyde 3-phosphate dehydrogenase (*GAPDH*) gene was used as an internal control to normalize the expressions of the mentioned interleukins. Then, changes in the expression levels of these genes were analyzed using real time-PCR method in a rotor gene (Qiagen, Hilden, Germany) detection system by utilizing SYBR GREEN^®^ Mastermix. The primer sequences used for the amplification of IL-5 and IL-13, are shown in Table 1.

**Histological evaluation**

The mice were euthanized using CO_2_ on the 31^st^ day; thereafter, the lungs were isolated and fixed by 10% neutral buffered formalin followed by embedding in paraffin. Hematoxylin and Eosin (H&E) and periodic acid Schiff (PAS) staining methods were performed to visualize the tissue sections. Mucus production was measured by visual scoring of the intensity of the PAS stain in 10 randomly selected microscopy fields on histological sections at 400 x magnifications in a way that no observable PAS stain was scored 0%. The number of goblet cells (PAS-positive cells) was quantified per 100 epithelial cells at several randomly selected microscopy fields at 400x magnification. GCI (Goblet Cell Index) was classified to 4 levels/scores as follows: score 0: GCI<5%, score 1: 5% ≤ GCI<25%, score 2: 25% ≤ GCI<50%, score 3: 50% ≤ GCI<75%, and score 4: 75% ≤ GCI ≤ 100% (Athari et al., 2016 ▶).

**Statistical analysis**

The resulting data which included data of BALF inflammatory cells, lung histopathology, and mucus hypersecretion in all groups (I-IV) were analyzed by one-way analysis of variance (ANOVA) or Turkey *post-hoc* test for comparing the four groups using SPSS software version 22. A significant difference was assumed wherever p<0.05.

## Results

**Total phenolics and total tannin contents**

The polyherbal combination contained phenolic and tannin compounds; therefore, in order to provide a marker for quality control of the product, total phenolic content and total tannin content were evaluated according to the British pharmacopeia method. Pyrogallol was used as the standard and the calibration curve was plotted (y = 4.4035x + 0.0398 R² = 0.9955). Total phenolics and total tannins were 1.850±0.09 and 0.706±0.08 g/100 g equivalent of pyrogallol for dried extract, respectively

**Thin layer chromatography**

**Figure 2:**
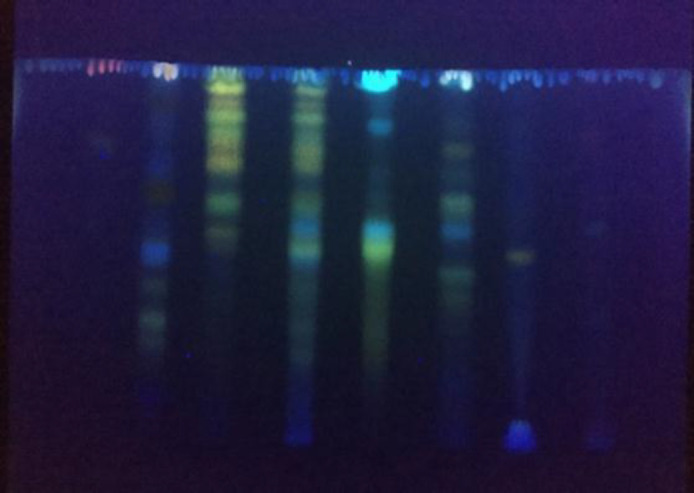
TLC fingerprint of “*Monzej-e-balgham*” and the corresponding ingredients. Stationary phase: silica gel; Mobile phase: ethyl acetate: formic acid: glacial acetic acid: water (100:11:11:26); Reagent: natural product; The fingerprint from left to right corresponds to *Adiantum capillus veneris*, *Glycyrrhiza glabra*,* Rosa damascena*, “*Monzej-e-balgham*”, *Onopordum acanthium*,* Pimpinella anisum*,* Ficus carica* and *Vitis venifera*

TLC of the polyherbal mixture and the ingredients is presented in Figure 2. The fingerprint of “*Monzej-e-balgham*” (the fourth) shows the spots corresponding to the major components.

**Infiltration of eosinophils into the BALF**

As shown in Table 2, there was a significant increase in eosinophil counts in BALF of OVA-sensitized group compared to the PBS-treated mice. “*Monzej-e-balgham*” treatment of the OVA-sensitized mice caused a significant decrease in the number of eosinophils in BALF samples of these animals. Budesonide, as a standard drug for the treatment of asthma, also had reducing effects on the number of eosinophils compared to the OVA-sensitized animals (p<0.05). Besides, BALF eosinophil decrease in the “*Monzej-e-balgham*” group was significantly different from the budesonide group (p<0.05). 

**Histopathological changes of the lung tissues**

**Figure 3:**
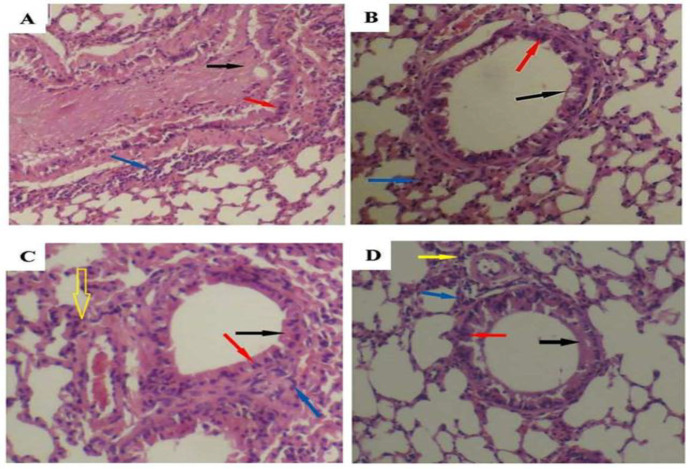
Effect of “*Monzej-e-balgham*” on allergic airway inflammation in lung tissues (H&E staining). (A) OVA; (B) Normal; (C) Budesonide and (D) “*Monzej-e-balgham*”. Arrows illustrate different cells; Black: mucus, Red: goblet cells, Yellow: perivascular inflammation, and Blue: peribronchial inflammation

Mucus secretion was significantly increased in the OVA-sensitized group compared to the PBS-administered controls (p<0.05). Our further analyses showed no significant difference in mucus secretion between the “*Monzej-e-balgham*” and budesonide groups (p>0.05) (Table 2, Figure 3). There was a considerable increase in the number of goblet cells in the OVA-sensitized animals in comparison to the PBS-treated mice (p<0.05). Goblet cell counts in the “*Monzej-e-balgham*” group were less than the budesonide group but similar to the PBS control group (Table 2 and Figure 3). Peribronchial and perivascular inflammations were higher in the OVA group compared to the PBS-treated mice and showed significant differences from both “*Monzej-e-balgham*” and budesonide groups (p<0.05). No difference was observed in this regard between the “*Monzej-e-balgham*” and budesonide groups (p>0.05). 

**Th-2 type cytokine levels in BALF specimens**

**Figure 4:**
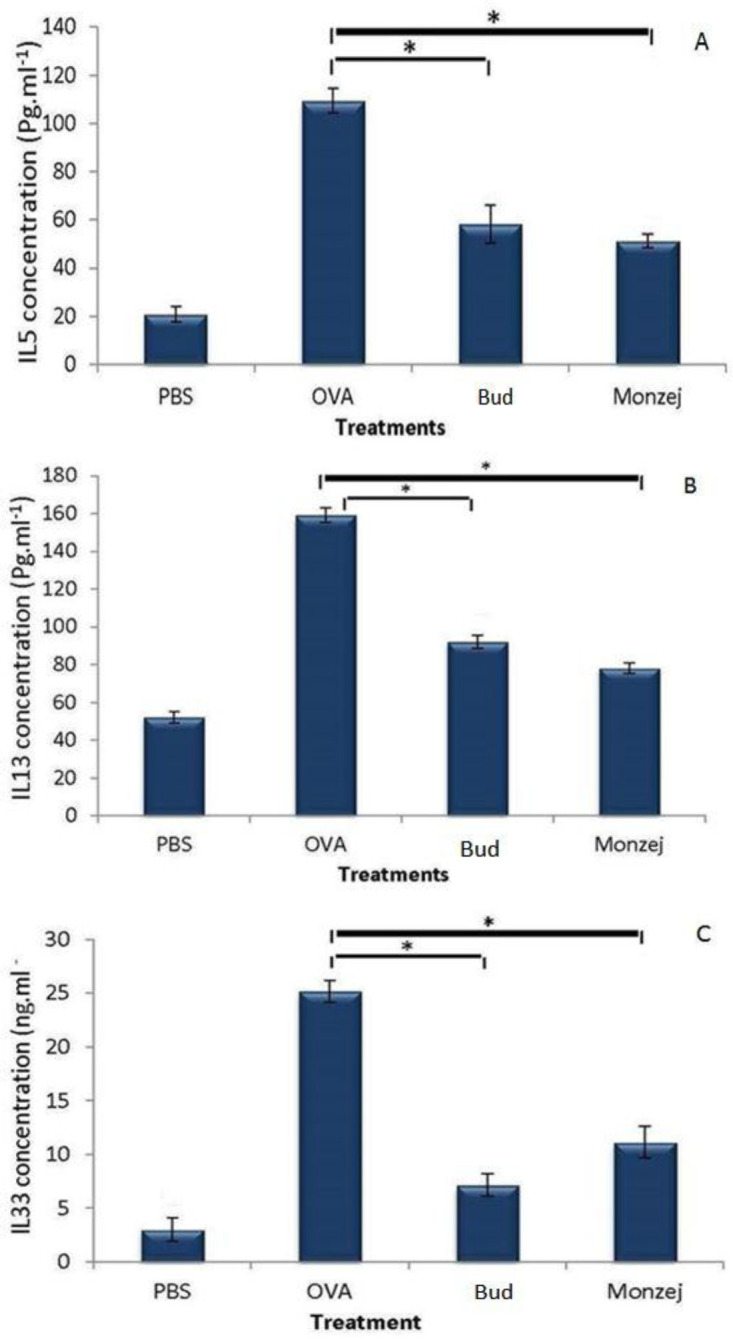
The concentrations of IL-5 (A), IL-13 (B) and IL-33 (C) in BALF samples obtained from different groups. Data is presented as Mean±SD; n=3, *indicates significant difference from the ovalbumin group (p<00.5)

The concentrations of the three Th2 type interleukins (i.e. IL-5, IL-13, and IL-33), were significantly increased in the OVA-sensitized group compared with the PBS-treated controls (p<0.05). In mice that received “*Monzej-e-balgham*”, the levels of these cytokines were remarkably reduced as compared with the OVA-sensitized mice. Moreover, the levels of IL-13 were significantly different between the “*Monzej-e-balgham*” and budesonide groups (Figures 4A, B and C). 

**Table 2:** Histopathological alterations of the lung tissues in different groups

	**PBS**	**Ovalbumin**	**“** ***Monzej-e-Balgham*** **”**	**Budesonide**
Mucus secretion (%)	25±5	100±5	25±10*	35±10*
Goblet cells	1.00±0.5	4.00±0.25	1.00±0.5*	1.30±0.7*
Peribronchial inflammation	0.50±0.2	4.00±0.25	1.5±0.2*	1.10±0.2*
Perivascular inflammation	0.50±0.1	4.00±0.25	1.00±0.5*	1.20±0.2*
EOS/BAL (%)	1.23	69.18	26.01*^#^	36.84*

Results are presented as mean±SD (n=3); *indicates significant difference from the ovalbumin group p<0.05; #indicates significant difference from the budesonide group p<0.05.

**Gene expression of IL-5 and -13 in BALF samples**

**Figure 5:**
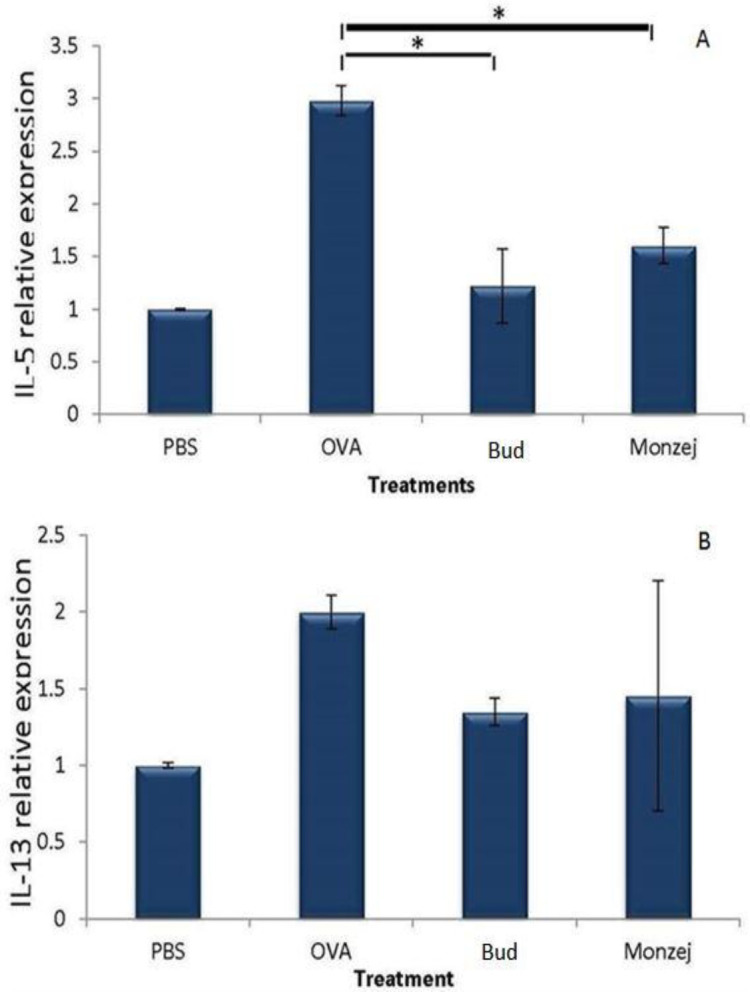
Gene expression levels of IL-5 and IL-13 following asthma induction and treatments. Data is presented as Mean±SD; n=3, *demonstrates significant between the treatment and ovalbumin groups (p<00.5)

The real-time PCR method was used to evaluate the alterations of the expression levels of both IL-5 and 1L-13 in BALFs of all groups. OVA sensitization of mice significantly amplified the gene expression of the mentioned interleukins compared with the PBS-treated negative control group (p<0.05). Figure 5A shows that in mice receiving “*Monzej-e-balgham*”, IL-5 expression levels were significantly reduced compared to the OVA-treated group. However, this polyherbal mixture non-significantly decreased the mRNA expression of IL-13 in comparison to the OVA-sensitized group. Budesonide treatment of mice had similar results to the “*Monzej-e-balgham*” group (Figures 5A and B). 

## Discussion

In this study, “*Monzej-balgham*” reduced goblet cells and decreased mucus content compared to the OVA-sensitized group. Moreover, increased levels of Th2 type cytokines due to OVA exposure, were significantly reduced by “*Monzej-balgham*”. The levels of IL-5, 13, and 33 were significantly decreased in the “*Monzej-balgham*”-treated mice when compared to the OVA-sensitized group. Interestingly, the mRNA expression of IL-13 had a non-significant reduction due to drug treatments but showed a significant decrease at protein levels. This indicates that regulation of this interleukin may be carried out at the protein level and its gene expression was not influenced by these treatments.

Cytokines are small glycoproteins produced by inflammatory cells in response to various stimulators, to mediate immunological responses (Arango Duque and Descoteaux, 2014). When a T-helper cell confronts an antigen, it differentiates to Th1 and Th2 which produce their specific cytokines. These two T subtypes can regulate the functions of each other (Sparano et al., 2004 ▶). Research on asthma patients and mice models of the disease has shown that activation of Th2 and production of Th2 type cytokines such as IL-13, IL-4, and IL-5, lead to activation of eosinophils and production of IgE. Thus, the ratio of Th2/Th1 may play an important role in triggering asthma (Lee et al., 2019 ▶; Liang et al., 2017 ▶). Guihua et al. demonstrated that eosinophil increase in BALF samples, as a sign of asthma, was significantly reduced by naringenin, a plant flavonoid (Guihua et al., 2016 ▶). IL-33 has been shown to potently activate the innate immune system and induce Th2 immune responses (Miller, 2011 ▶). IL-4 and IL-13 from Th2 cells activate B lymphocytes and increase the levels of IgE, which bond to the allergens and activate and degranulate mast cells, resulting in the release of inflammatory mediators including histamine, leukotrienes, prostaglandins, kinins, adenosines, nitric oxide, and cytokines (Deo et al., 2010 ▶; Millien et al., 2014 ▶). Mast cells are responsible for the initiation of airway contraction through releasing inflammatory mediators (Deo et al., 2010 ▶). On the other hand, IL-33 produced by alveolar type 2 cells can augment inflammation (Miller, 2011 ▶). It also affects Th2 cells, dendritic cells, mast cells, and eosinophils, which are involved in allergic disorders, to aggravate the inflammatory status of airways (Saikumar Jayalatha et al., 2021 ▶). The level of this interleukin is correlated with asthma severity (Lloyd and Saglani, 2015 ▶; Préfontaine et al., 2010 ▶) and has been found in bronchial biopsies of stable asthma patients (Porsbjerg et al., 2016 ▶). IL-33, along with IL-5 and IL-13, is involved in asthma complications and a positive correlation has also been reported between IL-33 levels and blood eosinophils count (Jackson et al., 2014 ▶). Moreover, increased secretion of goblet cells is stimulated by IL-4 and IL-13 which can elevate the amount of mucus in airways (White et al., 2010 ▶). Guihua et al. reported inhibition of extra mucus secretion in response to naringenin (Guihua et al., 2016 ▶). Choi et al. also revealed the decreasing effect of picroside-II (P-II), an iridoid glycoside derived from *Picrorhiza kurroa*, on the secretion of mucus into the airways (Choi et al., 2016 ▶). 

Effects of “*Monzej-balgham*” on OVA-induced allergic asthma in mice were evaluated in the present styudy. The results showed that “*Monzej-balgham*” reduced Th2 cytokines and eosinophil trafficking in BALFs of mice with allergic asthma. This shows that “*Monzej-balgham*” may be able to hamper the secretion of Th2 cytokines. This may hinder mucus-secreting cells to overproduce mucus into the airways and prevent the infiltration of eosinophils. It could be concluded that “*Monzej-balgham*” plays an important role in reducing inflammation in the respiratory tract and it may be suggested as a potential herbal remedy to treat allergic asthma-associated responses.

## Conflicts of interest

The authors have declared that there is no conflict of interest.
